# The derepression of transposable elements in lung cells is associated with the inflammatory response and gene activation in idiopathic pulmonary fibrosis

**DOI:** 10.1186/s13100-021-00241-3

**Published:** 2021-06-09

**Authors:** Mahboubeh R. Rostami, Martina Bradic

**Affiliations:** 1 Department of Genetic Medicine, Weill Cornell Medical College, 1300 York Avenue, Box 164, New York, NY 10065 USA; 2 Marie-Josee and Henry R. Kravis Center for Molecular Oncology, Memorial Sloan Kettering Cancer Center, New York, NY USA

## Abstract

**Background:**

Transposable elements (TEs) are repetitive sequences of viral origin that compose almost half of the human genome. These elements are tightly controlled within cells, and if activated, they can cause changes in both gene regulation and immune viral responses that have been associated with several chronic inflammatory diseases in humans. As oxidants are potent activators of TEs, and because oxidative injury is a major risk factor in relation to idiopathic pulmonary fibrosis (IPF), we hypothesized that TEs might be involved in the regulation of gene expression and so contribute to inflammation in cases of IPF. IPF is a fatal lung disease that involves the gradual replacement of the alveolar tissue with fibrotic scars as well as the accumulation of inflammatory cells in the lower respiratory tract. Although IPF is known to occur as a result of the complex interaction between age, environmental risk factors (i.e., oxidative stress) and genetics, the relative contributions of these factors to the disease remain unclear. To determine whether TEs are associated with IPF, we compared the transcriptional profiles of the genes and TEs of lung cells obtained from both healthy donors and IPF patients.

**Results:**

We quantified TE and gene expression levels using a published bulk RNA-seq dataset containing 24 subjects (16 donors and eight IPF patients), including three lung-cell types per subject, as well as an scRNA-seq dataset concerning 16 subjects (eight donors and eight IPF patients).

We found evidence of TE dysregulation in the alveolar type II lung cells and alveolar macrophages of the IPF patients. In addition, the activation of the LINE1 family of elements in IPF is associated with the increased expression of TE cellular regulators (MOV10, IFI16, SAMHD1, and APOBECG3), interferon-stimulating genes (ISG15, IFI6, IFI27, IFI44, and OAS1), chemokines (CX3CL1 and CXCL9), and interleukins (IL15RA). We also propose that TE derepression might be involved in the regulation of previously reported IPF candidate genes (MUC5B, CHL1, SPP1, and MMP7).

**Conclusion:**

Based on our findings, we propose that TE derepression plays an important role in the regulation of gene expression and can also prompt both the recruitment of inflammatory processes and the disruption of the immunological balance, which can lead to chronic inflammation in IPF.

**Supplementary Information:**

The online version contains supplementary material available at 10.1186/s13100-021-00241-3.

## Background

Over half the human genome is composed of repetitive sequences known as transposable elements (TEs). These repeated regions of the genome are organized into DNA transposons, which propagate via a cut and paste mechanism, and retrotransposons, which move by means of a copy and paste mechanism [1, 2]. Retrotransposons are the most prevalent TEs in humans, and they are further divided into the long terminal repeats (LTR) superfamily, which includes endogenous retroviruses (ERV), and the non-LTR superfamily, which includes both short interspersed elements (SINEs) and long interspersed elements (LINEs). Each of these superfamilies harbors a diverse family of repetitive sequences, and only the non-LTR elements from the L1, Alu, and SVA families can still transpose in the human genome [3]. TE transposition can destabilize the genome in many different ways, including gene disruption, the modulation of gene transcription, and mRNA processing through numerous mechanisms [4, 5]. Given their viral origin, the overexpression of TEs can also mimic viral infection and so trigger an innate immune response, leading to chronic inflammation [6, 7]. Although the TE activity is tightly regulated in somatic cells at the transcriptional and post-transcriptional levels, dysregulation can occur due to changes in DNA methylation, histone modification, or mutations in the genes involved in TE regulation [8–10]. Notably, elevated cytokine levels and chronic inflammation in response to increased TE expression have been previously associated with multiple human diseases, including multiple sclerosis, systemic lupus, lateral sclerosis, Rett syndrome, Aicardi-Goutières syndrome, aging-related pathologies and complex lung disorders [11, 12]. However, due to their repetitive nature, TEs are often excluded from analysis, which explains why their effect on expression and their involvement in the processes of diseases have not been systematically studied. Here, we aim to survey the TE activity in idiopathic pulmonary fibrosis (IPF) as well as to determine whether TE activation might be involved in both the IPF-related inflammatory response and the regulation of IPF-related genes.

IPF is a non-treatable inflammatory lung disease that involves the gradual replacement of the alveolar tissue with fibrotic scars as well as the accumulation of inflammatory cells in the lower respiratory tract [13]. The detection of IPF-associated genetic variants has enhanced our understanding of the role played by inherited risk factors in the disease risk. However, the underlying causes of IPF are not yet well understood and vital questions persist regarding how the complex interaction between risk factors (e.g., smoking, viral infection, oxidative stress, age) and genetics causes IPF pathogenesis [14]. An important feature of that pathogenesis is the shift in epithelial cell populations whereby type I alveolar (AT1) epithelial cells are damaged and the epithelial surface is populated by type II alveolar (AT2) epithelial cells and bronchiolar epithelial cells. The lower respiratory tract of an IPF patient is primarily populated by alveolar macrophages (AMs) and neutrophils, which are among the first responders to cellular defense and which play a significant role in absorbing harmful particles that have passed through the mechanical barrier of the respiratory system. When activated, AMs spontaneously release toxic oxidants (i.e., H_2_O_2_), which place a persistent oxidative burden on the fragile structure of the alveoli, and therefore, represent one of the most important mechanisms of AT2 epithelial cell injury in cases of IPF [15, 16].

As oxidants are potent activators of TEs [17], we hypothesized that exposure to oxidative stress fosters a permissive environment in lung cells that unleashes TEs, which modify the adjacent gene expression and also contribute to chronic inflammation due to their “viral mimicry” potential. To test this hypothesis, we used published transcriptome profiles of lung cells obtained from both healthy donors and pulmonary fibrosis patients [18]. This data were chosen because it allows for the profiling of the TE activity in the individual cell populations within those cells that are highly relevant to the disease. We determined the upregulation of TEs in IPF patients using the bulk RNA sequencing data in AT2, AM, and whole-lung cells, and we confirmed the upregulated TE activity of the L1 TE family in individual cell clusters using single-cell RNA sequencing (scRNA-seq) data. This is the first study to survey and relate TE activity to IPF. Additionally, it demonstrates that TE derepression might be involved in the regulation of previously reported IPF candidate genes, and further, that active TEs might be involved in the perpetual inflammation of the lower respiratory tract in cases of IPF.

## Results

### Increased TE expression is positively correlated with both the activation of cellular TE inhibitors and the innate immune response and negatively correlated with autophagy in IPF

To determine the expression of TE changes in IPF, we quantified the TE expression from previously published reports of the bulk RNA sequencing of 14 donor lung biopsies and compared it to explants from eight transplant recipients [18] (Supplemental Table 1). We first compared gene expression and expression of the TE families between IPF patients and donors in flow cytometry-sorted AMs, AT2, and whole lung cells (WLs). The TE families are defined as groups of TEs with similar sequences across the genome (subfamilies), and thus, their expression is averaged per the number of such groups.

**Fig. 1:**
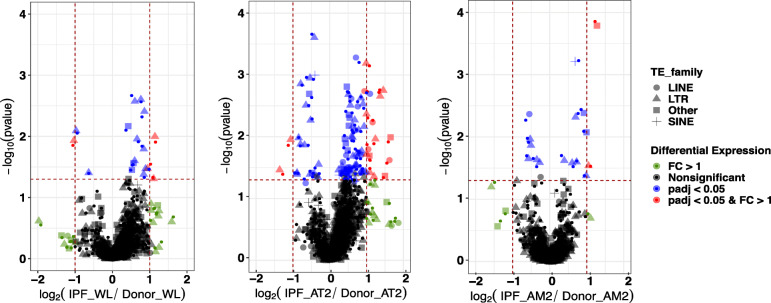
Changes in the expression of TE families in individual cell types and whole-lung tissue. **a** Bulk RNA-seq of whole-lung tissue (WL). **b** Bulk RNA-seq of flow cytometry-sorted alveolar type II cells (AT2). **c** Bulk RNA-seq of flow cytometry-sorted alveolar macrophages (AM). The TE expression was determined by read counts using the *SQuIRE* suite of tools, while the differential expression analysis was performed using the *DESeq2* package in R. The x-axis represents the log_2_ ratio of the TE subfamily expression between the IPF patients and the donors. The y-axis represents the adjusted *p*-value based on -log_10_. The red color represents the TE subfamilies with a fold expression change (FC) > 1 and p.adjust < 0.05 (as represented by two vertical dotted lines, and the horizontal dotted line). The blue color represents the TEs with an adjusted *p*-value (p.adjust < 0.05, BH adjusted), while the green dots represent the TEs with an FC > 1. The black color represents changes that do not have a significant p.adjust value or an FC higher than 1. Different shapes represent different TE families

**Fig. 2:**
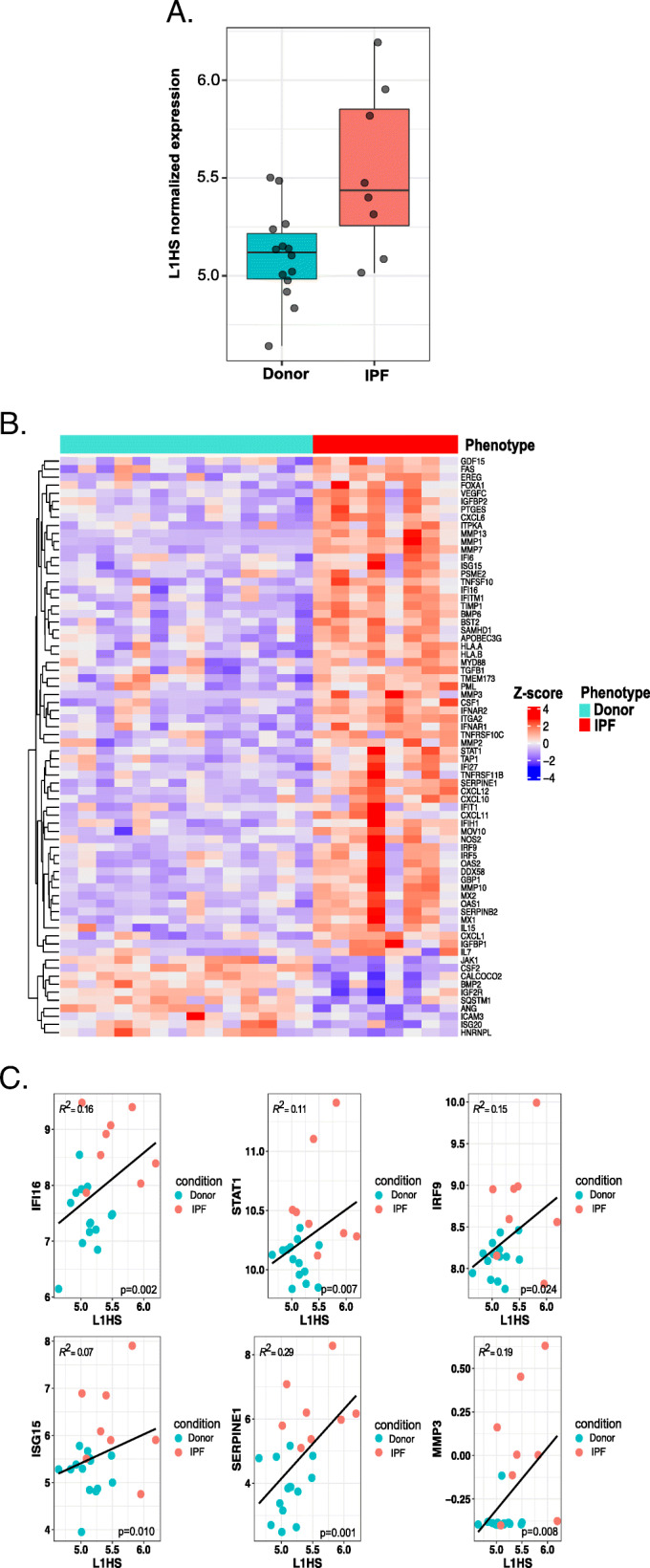
L1HS intergenic expression is associated with the IPF phenotype and expression of genes with functional relevance to L1HS activity in AT2 cells. **a** L1HS intergenic expression in Donor and IPF samples as quantified by *REdiscoverTE* method. Green: donor samples. Red: IPF samples. The x-axis represents groups; the y-axis represents log-normalized L1HS expression values. L1HS expression between the groups is significantly different as determined by *DESeq2* analysis (p.adjust = 0.001). **b** Heatmap of differentially expressed genes from four gene sets which are functionally relevant to L1HS activity [28, 29]: type I interferons (IFN-I, 33 out of 84 genes), senescence-associated secretory phenotype genes (SASP, 34 out of 85 genes), L1 transcriptional regulator genes (1 out of 2 genes), and L1 post-transcriptional regulator genes (6 out of 17 genes). The heatmap colors represent Z-score of log-normalized gene expression. The y-axis represents four gene sets. Samples are grouped on the x-axis by their phenotypes. **c** Examples of L1HS co-expression correlation with genes functionally relevant to L1HS activity from four gene sets: INF-I pathway (DDX58, IFI16, STAT1, IRF9, ISG15), senescence-associated secretory phenotype (SASP) genes (MMP3, SERPINE 1), L1 transcriptional regulator genes (FOXA1), and L1 post-transcriptional regulator genes (SAMHD1, MOV10, and APOBEC3G, CALCOCO2). Plots show the linear relationship of log-normalized gene expression which represents dependent variable (y-axis), and L1HS as an independent variable (x-axis) in the AT2 cells. Phenotype group (IPF/donor) was included as a covariate in the model. (Model: Gene expression∼L1HS expression + group (IPF/Donor). The colors represent the phenotypes (Green: donor; Red: IPF), and the gray line is the best fit from linear model. The R^2^ values from linear model fit of each gene-L1HS relationship are shown. The BH p-adjusted values (p) < 0.05) for each relationship are shown; p represents relationship between gene expression and L1HS expression. Results of all tested genes are summarized in Supplemental Table 4B-C

The largest changes in the expression of the TE family were identified in the AT2 cells (72 up, 22 down), with the largest number of changes being present in the LTR-TEs (Fig. 1, Tables 1, 2 and Supplemental Table 2 A-C). The WL that represents a mixture of different cell types exhibited 18 TE subfamily differences (14 up, 4 down), with the largest number of changes again being present in the LTR-TEs. Finally, the TE activity in the AMs was characterized by 17 TE subfamily differences (11 up, 6 down), again primarily in the LTRs. A few other TE expression changes representing DNA transposons, which are less abundant TE families in humans, were also identified. The changes in gene expression showed the same trend as the TE changes, and they were also the highest in the AT2 cells (4131), followed by the WL (1170) and AMs (1033) (Tables 1 and 2). We performed a Fisher exact test [19] to assess whether differentially expressed subfamilies are enriched in WL, AT2, or AM cells, and we did not find any significant enrichment (Table 2). Interestingly, the LINE elements, which are responsible for the majority of retrotransposition activity in humans, were only upregulated in the AT2 cells (Fig. 1, Tables 1 and 2). To confirm our findings, we also analyzed the data using *REdiscoverTE* [20], and verified the largest number of TE changes occurred in the AT2 cells with similar changes in the individual subfamilies (Supplemental Figure 1, Supplemental Table 2 D-G). AT2 cells are a primary target of injury in IPF, and the processes involved in AT2 cellular senescence and inflammation are crucial for the development of fibrotic lung disease [21–25]. Moreover, the LINE TE family, particularly the upregulation of the autonomous (expressed from intergenic regions, i.e., not from genic regions and thus not dependent on gene expression) L1HS subfamily, has a well-established impact on senescence-related inflammation [6, 7]. Thus, we first tested for any evidence of autonomous L1HS subfamily upregulation in the AT2 cells. To differentiate autonomous TE expression from co-expression with host genes or intron retention, we used *REdiscoverTE* [20]. This computational workflow is designed to separate reads at the family level according to their genomic location (intronic, exonic, and intergenic), and it specifically models autonomous TE expression. We observed the largest number of differentially expressed TE families in the IPF cells in the exonic region (65), followed by those in the intergenic (57) and intronic (32) regions (Supplemental Table 3A-D) if only the SINE, LINE, and LTR families were considered. Given the technical challenges with short sequencing reads to distinguish autonomous expression from host gene expression we limit our analysis to differential expression in the intergenic region [20, 26]. We detected overexpression of 16 LINE subfamilies, 38 LTR families, and three SINE families in the intergenic regions of the IPF cells (Supplemental Table 3C). These were all evolutionarily old TEs [27]; they were excluded from this particular analysis because their relationship with the inflammation during cellular senescence is functionally not well-established. Importantly, the L1HS family that represents human specific and retrotransposition competent TE element was significantly upregulated only in the intergenic region of the IPF cells (Fig. 2a, Supplemental Table 3C) [30]. This suggests autonomous L1HS expression that could be related to the cellular processes involved in IPF. A similar pattern of differential intergenic TE expression in AT2 was observed using the modified *SQuIRE* quantification method (see Materials and Methods for details) (Supplemental Table 3D and Table 3E). The L1HS expression between groups as quantified by the modified *SQuIRE* quantification method was only significantly different prior (*p* = 0.037, logFC = 1.16), but not after multiple test correction (BH p.adjusted = 0.11). (Supplemental Figure 2). This discrepancy is a result of different mapping and quantification methods used by *SQuIRE* and *REdiscoverTE.*

**Table 1:** Differential expression analysis of TEs families and genes in three cell types. Number of significant changes (p.adjusted < 0.05) between the donors and IPF patients in the WL, AT2 and AM are shown

	WL	AT2	AM	
TE Family	UP/DOWN	UP/DOWN	UP/DOWN	
SINE	0/0	1/1	1/0	
LINE	0/4	17/2	0/1	
LTR	11/0	40/17	6/5	
Other	3/0	14/2	4/0	
**Genes**	654/516	2477/1654	566/467	

The TE changes are shown at the family level whereas each number represents number of TEs subfamily changes. *WL* whole lung, *AT2* alveolar type II cell, *AM* alveolar macrophage

**Table 2:** Enrichment analysis of individual elements in three cell types. Significant changes (p.adjusted < 0.05) between the donors and IPF patients in the WL, AT2 and AM are shown

	Differential expression	No differential expression	Total in the genome	Fisher exact test of enrichment
**WL**				
LINE	4	179	183	0.75
LTR	11	577	588	0.75
SINE	0	63	63	0.61
**AT2**				
LINE	19	164	183	0.56
LTR	57	531	588	0.69
SINE	2	61	63	0.11
**AM**				
LINE	1	182	183	0.31
LTR	11	577	588	0.36
SINE	1	62	63	1.00

The TE changes are shown at the family level whereas each number represents number of TEs subfamily changes. *WL* whole lung, *AT2* alveolar type II cell, *AM* alveolar macrophage. Enrichment analysis of individual families was performed using Fisher exact test where each family was tested against the sum of other families for each cell type. BH adjusted *p*-values are shown. Only LINE, SINE and LTR families were considered

AM and WL cells were also inspected for L1HS intergenic expression, and no differential expression was detected between the IPF and donor cells (data not shown).

TEs are tightly controlled by multiple mechanisms, including TE promoter methylation, and the inhibitory host factors involved in transcriptional and post-transcriptional TE control [31]. Modifications in these control points might activate TEs and cause pathological states that are frequently accompanied by inflammation [6, 11, 20, 32]. However, cellular transformation to a disease state in IPF is also complemented by these processes [21, 22, 33]. Thus, we determined whether there was an association between L1HS upregulation, and the expression of genes with functional relevance to L1HS activity [31, 34]. We tested four gene sets from the published literature: type I interferons (IFN-I, 84 genes), senescence-associated secretory phenotype (SASP) genes (85 genes), L1 transcriptional regulator genes (two genes), and L1 post-transcriptional regulator genes (17 genes) using linear model.

A total of 73 of these genes were differentially expressed between the IPF and donor cells; 63 were upregulated and ten were downregulated out of the 188 genes from our four sets (Fig. 2c and Supplemental Table 4B-C). Our linear model analysis showed a significant association between L1HS and 60 genes that differed between the IPF and donor cells (Supplemental Table 4C)*.*

Here, we highlighted a correlation between L1HS and a few well known functionally important genes. We observed a positive association between L1HS and the forkhead box A1 (FOXA1) transcription factor (TF), which is crucial for L1HS expression; this finding is further evidence of the active expression of L1HS in the IPF cells (Fig. 2b and c, Supplemental Table 4C) [6]. However, post-transcriptional regulator CALCOCO2 (NDP52), which plays an important role in degrading L1 RNA in the cytoplasm [35], was downregulated in the IPF patients and negatively correlated with the expression of L1HS (Fig. 2b and c, Supplemental Table 4C). This might suggest an uncontrolled accumulation of L1HS in the IPF cells. Genes involved in IFN-I response (e.g., DDX58 (RIG-I), IFI16, STAT1,IRF9, ISG15), which is important for recognition of L1 cytosolic accumulation, and downstream activation of inflammatory processes also showed positive correlations with L1HS [31] (Fig. 2b and c, Supplemental Table 4C). This further suggests that the inflammatory processes in IPF cells are related to increased L1HS expression.

We also tested genes that act as L1HS post-transcriptional regulators and found positive correlations for three of them in the IPF cells (Fig. 2c [SAMHD1, MOV10*,* APOBEC3G], Supplemental Table 4-C) [31]. Some of these L1 inhibitors(i.e., SAMHD1, MOV10*,* and APOBEC3G) are activated by IFNs [28]. Thus, increased interferon signaling may have activated these L1 inhibitors against the high L1HS expression in the AT2 cells.

Finally, SASP markers (e.g., SERPINE1 and MMP3) were also positively associated with L1HS in the IPF cells [6] (Fig. 2c, Supplemental Table 4-C). This is particularly interesting, as previous research showed increased SERPINE1 in IPF AT2 senescent cells [36]. Increased MMP3 expression is also essential for the pathogenesis of IPF, based on findings from IPF patients and animal models [37]. We found extremely low MMP3 expression in the donor cells, whereas the IPF cells expressed high levels of MMP3, which was positively associated with L1HS. This further suggests that senescent cells in IPF patients also exhibit an increase in L1HS activity.

### Locus-level TE expression is associated with IPF and is also cell-type specific

The induction of immune response in cellular senescence by means of the accumulation of the intrinsically expressed TEs in the cell is not the only detrimental effect of TEs. Indeed, TEs can provide alternative promoters or polyadenylation signals, as well as alternative splice acceptor and donor sites, which can strongly alter the gene expression patterns of the host [5, 38]. In addition, a few methods based on the association between TE and proximal gene expression have been developed as potential tools for detecting candidate genes for diseases [39].

**Fig. 3:**
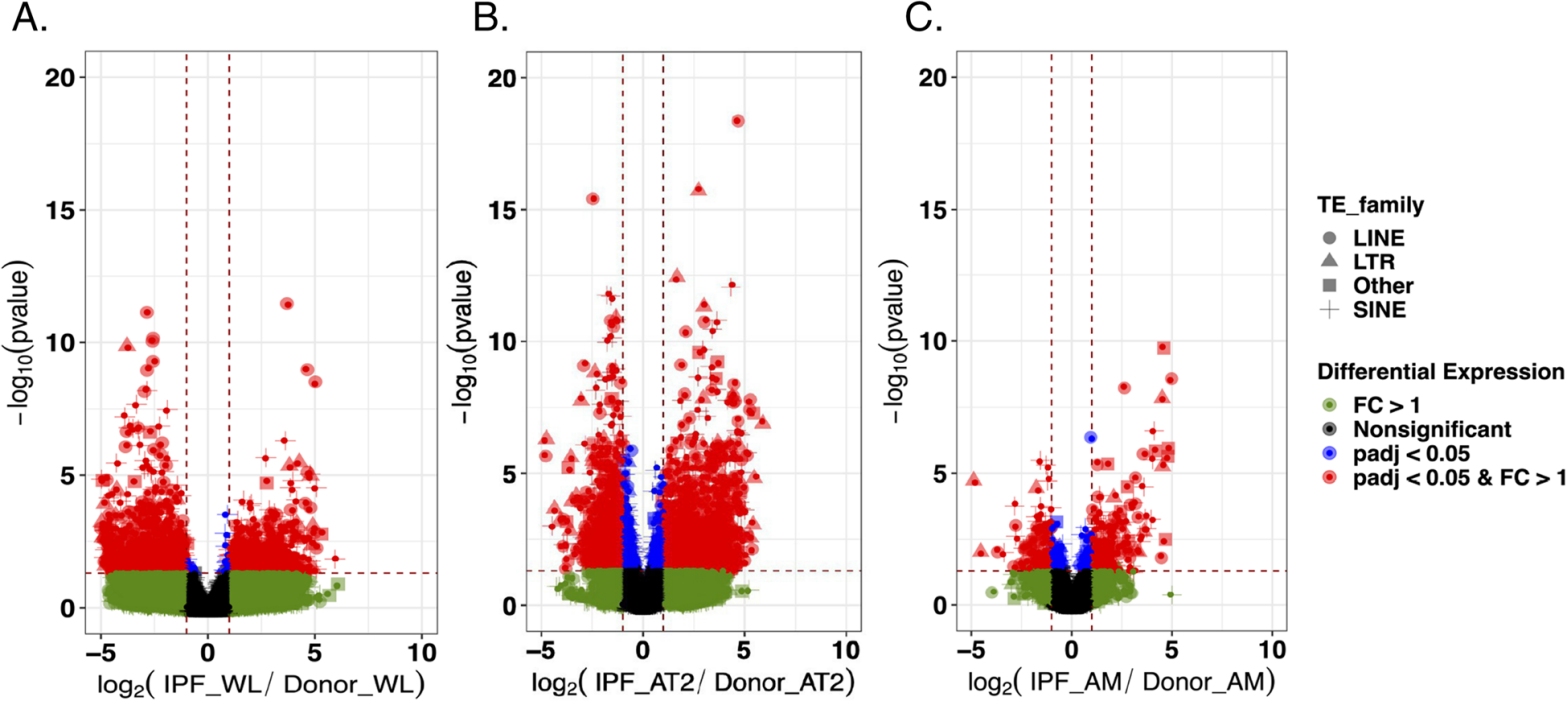
Differentially regulated TE loci in IPF patients vs. donors in cell types and whole-lung tissue. **a** Bulk RNA-seq of whole-lung tissue. **b** Bulk RNA-seq of flow cytometry-sorted alveolar type II cells (AT2). **c** Bulk RNA-seq of flow cytometry-sorted alveolar macrophages (AM). The TE expression was determined by read counts using the *SQuIRE* suite of tools, while the differential expression analysis was performed using the *DESeq2* package in R. The x-axis represents the log_2_ ratio of the TE locus expression between the IPF patients and donors. The y-axis represents the adjusted *p*-value based on -log_10_. The red color represents the TE loci with a fold expression change (FC) > 1 and p.adjust < 0.05 (BH adjusted) (as represented by two vertical dotted lines and horizontal line). The blue color represents the TEs with an adjusted *p*-value (p.adjust < 0.05), while the green dots represent the TEs with an FC > 1. The black color represents changes that do not have a significant p.adjust value change or an FC higher than 1. Different shapes represent different TE families

**Fig. 4:**
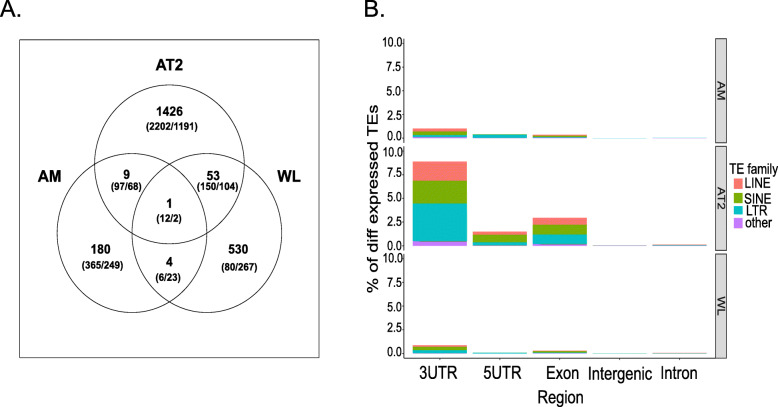
Summary of the IPF-associated TE loci changes and their association with the adjacent genes. **a** A Venn diagram of the overlap between the upregulated TE loci in the IPF patients when compared with the donors in whole-lung tissue (WT), alveolar type II cells (AT2), and alveolar macrophages (AM) (upper number). An overlap between the upregulated (first number in the brackets) and downregulated genes (second number in the brackets) is also shown. **b** Histograms summarizing the TE expression changes by intergenic and intragenic regions in the WL, AT2 and AM. The intragenic regions are divided into the 3’UTR, 5’UTR, intron, and exon regions. The different colors represent the proportion of each TE family in each region. The x-axis represents the individual genomic region. The y-axis represents the percentage of TE loci per TE family that are normalized by the total number of each TE family present in each genomic region

Here, we aim to determine whether locus-specific TE expression near or within the gene can explain the expression differences of that gene, and whether that relationship differs between donor and IPF cells. We quantified the changes in TE expression that occurred at individual genomic locations for all TE families within the three cell types, and associated those differences with adjacent gene expression. We again identified the largest number of changes in the AT2 cells (1489 up, 1149 down), while the WL also featured an abundant number of changes representing 1341 TE loci (588 up, 753 down) (Fig. 3, Supplemental Table 5). Finally, the TE activity in the AM was found to be characterized by 359 TE loci (193 up, 166 down). We first intersected the upregulated TE locus changes as well as the gene changes between the IPF patients and the healthy donors in each cell type (Fig. 4a). This analysis revealed that most changes were unique and only present in an individual cell type (AT2 = 1426, AM = 180, and WT = 530), although 53 TE changes were shared between the AT2 and WL, four between the WL and AM, and nine between the AM and AT2, while one change was common to all three comparisons. This suggests the potentially strong impact of TE activity specific to the AT2 and AM cells that might affect the gene expression related to IPF and that is not detected when analyzing the heterogeneous WL cell population. The number of gene expression changes shared among the different cell types also showed a similar trend, with the highest number of expressed genes being observed in the AT2 (3826), followed by the AM (822), and the WT (644) (Fig. 4a).

We next examined the distribution of the TE changes across the genomic regions. To do so, all the differentially expressed TE changes were categorized as belonging to either the 5’UTR, 3’UTR, exon, intron, or intergenic region. These analyses revealed the same pattern of changes as observed in relation to the three comparisons (AT2, AM, and WL, Fig. 4b, Supplemental Table 5), with the highest number of TE transcriptional changes being seen in the 3’UTR region, while the smallest number of changes were found in the intron and intergenic regions. The TE changes that overlap with the exon as well as the exon-intron, exon-5’UTR, and exon-3’UTR regions were excluded from further analysis because they were most likely related to transcriptional noise.

### TE expression is correlated with the gene expression of several IPF candidate genes

**Fig. 5:**
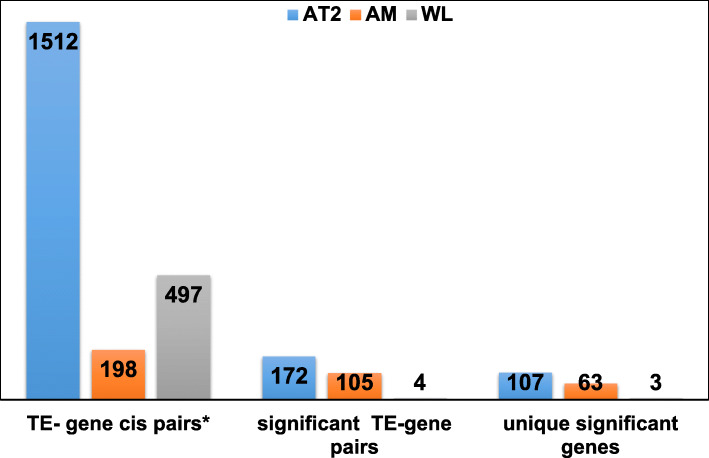
Barplots summarizing the TE-gene associations in the whole-lung tissue, AT2, and AM. The association between the log_2_-transformed TE expression (independent variable) and the log_2_-transformed gene expression (dependent variable) was tested using a linear model (*lm*). Phenotype group (IPF/donor) was included in the model as a covariate. The *p*-value correction was performed using the Benjamini and Hochberg (BH) test. Only the expression of significant genes and significantly upregulated TEs located next to the gene (max distance 50 kb, cis loci) are summarized here (p.adjusted < 0.05)

To further illustrate the importance of locus-specific TEs, we tested the effects of the intragenic and intergenic upregulated TE (excluding those in the exons) loci on the transcriptional regulation of the genes. As the pairwise comparisons of each gene with each TE locus identified a large number of changes, some of which proved difficult to interpret, we focused on the TEs matched with their adjacent genes (cis interactions) and examined whether TE expression predicts nearby gene expression. There were 1512 pairs (out of 10,092,988 tests conducted between 3826 genes and 2638 TEs, with 7.80% being significant pairs) in the AT2 cells, 198 pairs in the AM cells (out of 295,098 tests conducted between 822 genes and 359 TEs, with 1.4% being significant pairs), and 497 pairs in the WL (out of 863,604 tests conducted between 644 genes and 1341 TEs, with 0.04% being significant pairs) that matched the cis criteria (Fig. 5, TE- gene cis pairs*). We identified a total of 172, 105, and four significant gene-TE pairs (*p* < 0.05) from our cis subsets in the AT2, AM2, and WL, respectively. In some cases, there were more than one TE associated with the expression of the same gene. Thus, we further classified the difference into the number of unique genes whose transcription is significantly related to the TEs. This classification resulted in the identification of a total of 107 genes in the AT2 cells, 63 genes in the AM cells, and three genes in the WL cells that had a transcriptional pattern correlated with TEs and that differed between the IPF patients and the healthy donor (Fig. 5). Interestingly, although a high number of TE-gene pairs were present in the WL comparison, only a very few significant differences were observed (3), which again suggests that important signatures might be missed in heterogeneous cell populations. Our gene ontology enrichment analysis of the genes found to be correlated with TE expression in the AT2 cells identified cilium movement as well as axoneme and organelle assembly related processes (FDR < 0.05) (Table 3). This suggests that the activation of TEs can result in the activation of genes that might be involved in cellular identity changes. In the AM cells, the TE-associated gene expression was found to be linked with immune-related cellular process.

**Table 3:** Genomic distribution and gene ontology enrichment of genes significantly associated with TEs in the AT2 and AM cells. A gene ontology (GO) enrichment analysis was performed on those genes with significant cis TE associations using the STRING database [40]. The total number of observed and background genes in each GO term category as well as all false discovery rate values (FDR) < 0.05 are shown for the AT2 and AM cells

GO TERM	Description	Observed gene count	Background gene count	FDR
**AT2**				
GO:0035082	axoneme assembly	6	59	0.002
GO:0003341	cilium movement	5	61	0.009
GO:0060271	cilium assembly	10	326	0.009
GO:0070286	axonemal dynein complex assembly	4	32	0.009
GO:0120031	plasma membrane bounded cell projection assembly	11	413	0.009
GO:0036159	inner dynein arm assembly	3	16	0.026
GO:0070925	organelle assembly	12	666	0.044
**AM**				
GO:0006955	immune response	16	1560	0.016
GO:0045124	regulation of bone resorption	4	38	0.016
GO:0002376	immune system process	19	2370	0.028

**Fig. 6:**
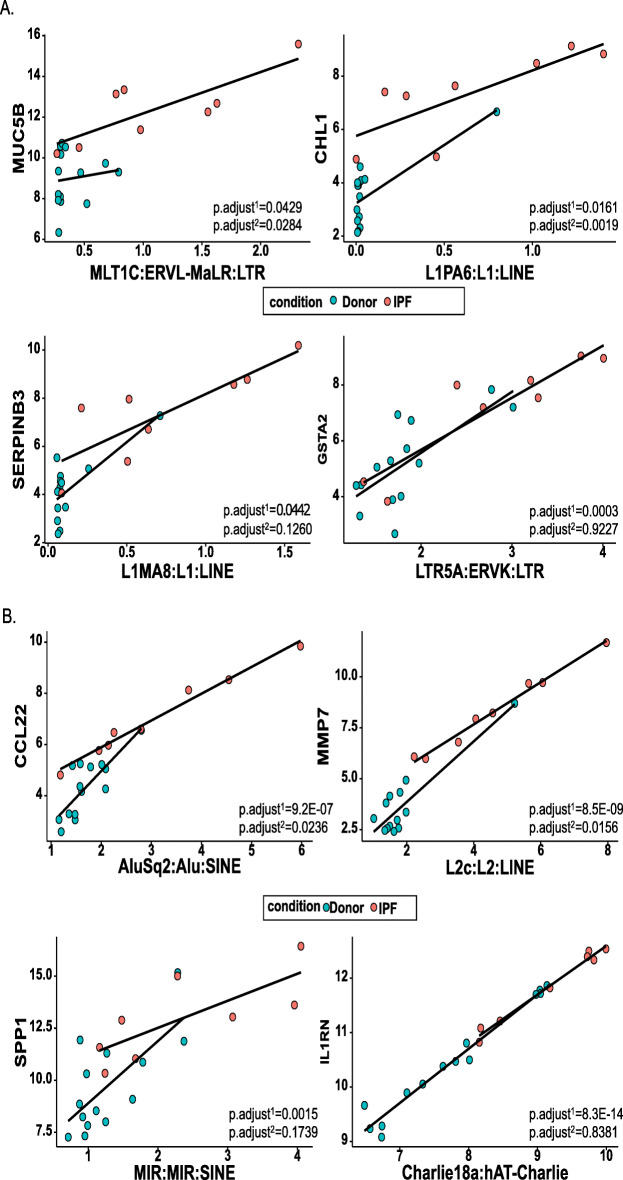
Examples of the gene expression correlation with the adjacent TE loci. **a** Correlation plots representing the correlation between the MUC5B, CHL1, SERPINB3, and GISTA2 genes and the adjacent TE loci in the AT2 cells. **b** Correlation plots representing the correlation of the CCL2, MMP7, SPP1, IL1RN genes and the TEs in the AM cells. The x-axis represents the normalized expression values for the TEs, while the y-axis represents the normalized expression values for the genes. The colors represent the phenotypes (Green: donor; Red: IPF patient). The BH p.adjusted (p.adjust < 0.05) values for each relationship are shown; p.adjust^1^ represents relationship between gene expression and adjacent TE-locus expression, and p.adjust^2^ represents difference in the regression slopes between the two groups (IPF-red, and donor-green)

Most of the TEs found to be associated with gene regulation in the AT2 cells were identified in the introns (75), followed by the 3’UTR region (82), outside the gene (12), and the 5’UTR region (3) (Supplemental Table 6A, Table 4). The SINE (Alu) elements were most commonly found to be associated with gene expression in the AT2 cells, and they were mostly embedded within the 3’UTR region of the genes. Several of the genes found to be significantly correlated with TEs represent important IPF candidate genes that have been identified in multiple genome-wide association studies or functional studies related to IPF [41]. For example, we identified a 3.88-fold expression change between the IPF patients and the healthy donors in the TE (chr11|1,253,519|1,253,937|MLT1C:ERVL-MaLR:LTR) that is located between exons 33 and 34 and that is significantly correlated with the high expression of the MUC5B gene in IPF (Supplemental Table 6A). Furthermore, the relationship between the MUC5B and the TE expression of IPF group is significantly different than the same relationship in donor group (Fig. 6a, p.adjust = 0.0284). This suggests the potential role played by TEs in the regulation of this gene, which in turn plays an important role in mucin excretion and significantly contributes to IPF pathogenesis [42, 43]. Notably, we identified the association between one of the three core IPF gene markers, namely the cell adhesion molecule L1-like (CHL1) gene, and the L1PA6 TE (chr3|367,661|374,053|L1PA6:L1:LINE) (Supplemental Table 6A). However, two other TEs (chr3|391,232|391,535|AluSz:Alu:SINE, chr3|408,347|408,547|MIRc:MIR:SINE|342), also showed a significant association with CHL1 expression. To determine which of the TEs made the largest contribution to CHL1 gene expression, we used all three TEs in a linear model and then calculated the relative importance of each of them in terms of influencing CHL1 expression. The proportion of the variance explained by the model containing all three TEs was 86.09%. Further, each TE explained ~ 20% of the variance, while the group covariate representing the phenotype explained ~ 25% of the variance, thereby suggesting similar contributions of each TEs to CHL1 gene expression. Significant difference between the correlation slope in IPF and donor groups was also observed for L1PA6- CHL1 relationship (Fig. 6a, p.adjust = 0.0019) and the other two TEs (AluSz, MIRc) associated with CHL1. This further suggests potential regulation of CHL1 by adjacent TEs in IPFs.

**Table 4:** Summary of genomic distribution of TE-gene cis loci pairs in AM and AT2 cells identified by linear model test. Number of significantly observed changes (p.adjsuted < 0.05, BH adjusted) are shown per each genomics region

TE family	Intron	Intergenic	3’UTR3	5’UTR
**AT2**				
SINE	30	5	38	1
LINE	26	3	26	0
LTR	14	4	11	1
Other	5	0	7	1
**AM**				
SINE	7	0	46	3
LINE	4	0	22	0
LTR	1	0	5	0
Other	1	0	14	1

The serpin family B member 3 (SERPINB3) is another important IPF candidate gene for which we identified a 4.23-fold change in the gene expression between the IPF patients and the healthy donors, which was correlated with a 4.21-fold change in the LINE TE element (chr18|63,651,629|63,653,187|L1MA8:L1:LINE) (Supplemental Table 6A). This element is located ~ 2 kb downstream of the gene. Glutathione S-transferase alpha 2 (GSTA2) also exhibited upregulation in the IPF patients, and its expression was associated with the LTR5A TE (chr6|52,748,278|52,749,305|LTR5A:ERVK:LTR) located 783 bp downstream of that gene (Fig. 6a, Supplemental Table 6A). Although TEs significantly predicts the expression of SERPINB3 and GSTA2 genes, that relationship does not significantly differ between IPF and donors as shown by test for correlation slopes (Fig. 6a, p.adjust = 0.1260, p.adjust = 0.9227, respectively). Nevertheless, significant difference of TE expression results in significant difference in gene expression for these genes in IPF suggesting their role in the disease.

The analysis of the TE loci in the AM cells primarily determined the association between gene expression and the SINE elements (56), followed by the LINE (26), LTR (6), and 17 other elements (e.g., DNA transposons) (Supplemental Table 6B). Most of the elements from all the families were activated within the 3’UTR region, and we did not identify any elements associated with gene expression outside the gene (Table 4). We found that the expression of the chemokine CCL22 was related to the three SINE elements (chr16|57,364,478|57,364,778|AluSq2:Alu:SINE, chr16|57,364,806|57,365,101|AluSz:Alu:SINE, chr16|57,365,617|57,365,740|AluJo:Alu:SINE) located in the 5‘UTR and 3‘UTR region (Supplemental Table 6B). This chemokine contributes to activation of alveolar macrophages and subsequently to the lung damage in patients with IPF [44]. The proportion of variance explained by the model predicting the CCL22 expression was 93.71%, with AluSz (3’UTR) explaining ~ 29% and AluJo (3’UTR) and AluSq2 (5’UTR) explaining ~ 27% and ~ 24%, respectively. Correlation slopes for IPF and donors are also significantly different for two of these elements providing additional evidences for strong regulation of CCL2 by TEs activation in IPF patients (Fig. 6b, only plot for AluSq2 element is shown, p.adjust = 0.0237). We also found that the upregulated TE expression in the intron (chr14|22,843,913|22,844,208|AluSx1:Alu:SINE), and in the 3‘UTR region (chr11|102,520,549|102,520,704|L2c:L2:LINE) significantly correlates with two upregulated matrix metalloproteases (MMP14 and MMP7, respectively) in IPF group (Supplemental Table 6B). MMPs are important players in cell migration and tissue repair in lungs and have been related to IPF pathogenesis [45]. Here, we establish correlation of two MMPs with TEs and we further identify significant TE-gene correlation slope difference in MMP7 between IPF and donors which further confirms differential regulation of MMP7 in IPF (Fig. 6b, p.adjust = 0.0156).

We were also able to associate a number of other genes with TE expression that have previously been related to IPF, including osteopontin (SPP1) in the 3’UTR region (Chr4: 87979662–87,979,893) and the interleukin 1 receptor antagonist (IL1RN) within the 5’UTR region (chr2|113,133,292|113,133,561|Charlie18a:hAT-Charlie:DNA) (Supplemental Table 6B). Both these genes are known to be expressed at high levels in IPF AM cells, while previously reported immunohistochemistry results have confirmed that these markers are not expressed in donor tissue [18]. Our study also finds significant upregulation of these genes and their adjacent TE loci in IPF.

However regression slopes between donors and IPF did not differ for these two TE-gene pairs, suggesting that these genes might not be directly related to TE expression (Fig. 6b, p.adjust = 0.1739 for SPP1, p.adjust = 0.8381 for IL1RN) .

### ScRNA-seq analysis confirms the TE changes in multiple cell populations in the fibrotic human lung

**Fig. 7:**
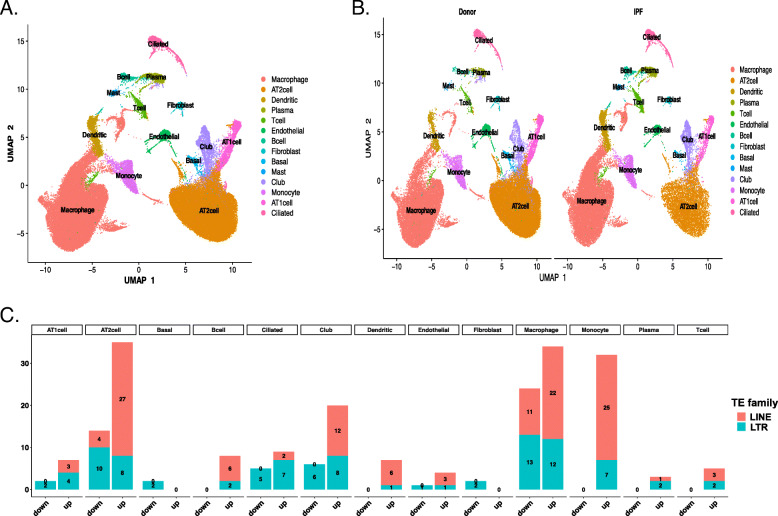
ScRNA-seq analysis summary. **a** Visualization of the different cell types in a uniform manifold approximation and projection (UMAP). **b** UMAP representation of the cells from donor (eight samples) and IPF (eight sample) patients. **c** The number of the significantly dysregulated TE subfamilies (p.adjust < 0.05) in eight fibrosis patients vs. eight donors in different cell types. A total of 77,517 single cells were profiled with scRNA sequencing. The analyses were performed using the *Seurat* (v3) R package. The cells were clustered using a graph-based shared nearest neighbor clustering approach, and 14 cell types were identified

We also analyzed a previously published dataset concerning eight donors and eight IPF patients that had been generated by means of scRNA-seq technology [18]. A total of 77,517 single cells and 22,009 genes were obtained. We assigned each cluster to a cell type based on the expression of the established markers in that cluster (Supplemental Figure 3), and the following cell types were confirmed: epithelial cells (alveolar type II cells (AT2), alveolar type I cells (AT1), ciliated cells, basal cells, and club cells), immune cells (alveolar macrophages (AMs), monocytes, B cells, plasma mast cells, dendritic cells, and T cells), and mesenchymal cells (fibroblasts and endothelial cells) (Fig. 7a). The distribution and identity of the cells were similar between the two phenotypes (Fig. 7b, Supplemental Table 5A). To confirm the differential TE activity in the AM2 and AT2 cells in the IPF patients, as well as to determine whether the TEs differed in the other cell types, we performed a differential gene expression (DGEs) analysis between the donors and the IPF patients with regard to the TE subfamilies in each cell type (Supplemental Table 7B). The results of the DGEs analysis between the IPF patients and the donors for each identified TE subfamily and all the genes in the individual lung cell type are presented in Supplemental Table 7C and summarized in Fig. 7c. We identified the increased transcription of two TE families (LINE and LTR) in the IPF patients when compared with the donors in multiple cell types (p.adjust < 0.05 and lfc > 0). Consistent with the TE upregulation identified in our RNA bulk analysis, we identified 27 significantly upregulated L1 subfamilies and eight significantly upregulated LTR subfamilies in the single-cell transcriptome. Similarly, 22 LINE subfamilies and 12 LTR subfamilies were found to be upregulated in the AM cells (Fig. 7c). In addition, we also detected upregulation in the case of the IPF patients in the monocytes as well as the upregulation of a smaller number of subfamilies in the club, ciliated, B, dendritic, AT1, T, endothelial, and plasma cells.

**Fig. 8:**
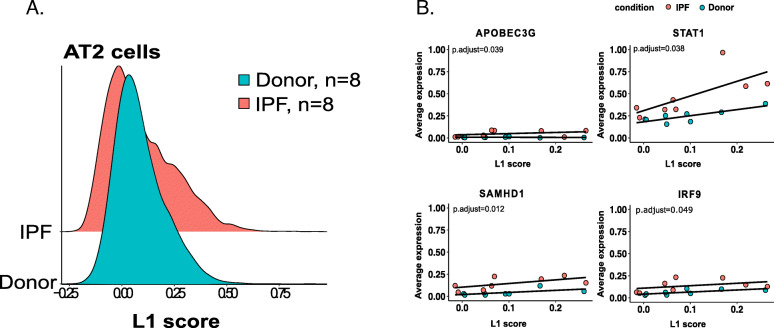
Differential L1 score distribution in the AT2 cells and its association with cellular factors. **a** The L1 score differences in the AT2 cells from eight fibrotic (red) vs. eight donor (green) lungs (*p* = 1.18-e09, *n* = 8 per phenotypic group). The L1 scores were calculated per each cell using all the expressed L1 elements, and the significance of the L1 score distribution between the phenotypes was determined using the Wilcoxon rank-sum test. The calculations were performed using the *Seurat* (v3) R package. **b** Correlation plot showing the relationship between the TE L1 score and the individual TE-related genes in the AT2 cells. The difference in the gene expression response to the L1 score in the AT2 cells was modeled using the *lm* function in R, the TE L1 score (independent variable), and the log_2_ transformed gene expression (dependent variable). To determine whether the relationship between L1 score and average gene expression differs between IPF and donors we used t-test. The *p*-value was corrected for multiple tests using the Benjamini and Hochberg (BH) test. Significant *p*-values (p.adjust < 0.05) are shown

Aside from identifying changes in multiple subfamilies, we were also interested in determining whether an association exists between the total expression of the L1 subfamilies and IPF in AT2 cells, as identified by bulk RNA-seq. Thus, we calculated the L1 score that represents the average expression of the upregulated L1 family per each cell type. The comparison between the L1 scores of the healthy donors and the IPF patients indicated that the L1 score is significantly higher among fibrosis patients in the AT2 cells, which indicates that it might be involved in IPF pathogenesis (Fig. 8a). To further confirm whether the relationship between L1 activity (L1 score) and TE-related genes differs among the IPF patients and the healthy donors, we tested 123 expressed genes out of 188 genes from four gene sets. We identified 27 significant TE regulation and inflammatory response related genes (p.adjust< 0.05) (e.g., APOBE3G, STAT1, SAMHD1, IRF9) in the AT2 cells (Supplemental Table 7D, Fig. 8b). This observation indicates that the L1 upregulation, also confirmed in independent dataset by scRNA-seq might promote the inflammatory response in the AT2 cells of IPF patients. Single-cell RNA sequencing is a powerful method but only generates short reads from one end of a cDNA template, limiting the mapping of highly similar TE sequences. Thus, locus specific TE expression was not performed for scRNA seq dataset.

## Discussion

### TEs and inflammation in IPF

The derepression of TEs can cause changes at the transcriptional and post-translational levels that involve gene expression changes, and further, that recruit immune signaling pathways that might result in pathologies [5, 46–48]. Our study suggests potential involvement of TEs in IPF, and we find evidence that TEs are significantly upregulated in the AT2 cells of fibrotic lungs. Aside from the overexpression of TEs in AT2 IPF cells, we also find that the dysregulation of TEs is likely further facilitated by the decreased expression of the autophagy gene CALCOCO2 (NDP52), which is known to be crucial receptor for the detection and removal of at least one TE RNA family (L1). Dysfunctional autophagy has also been previously associated with IPF, with the suggestion being that it promotes the epithelial–mesenchymal transition of the AT2 cells contributing to fibrosis [49]. Moreover, the failure of the autophagosome removal of TEs leads to cytoplasmic TE accumulation, which can result in genome instability and inflammation [35] and, in turn, can contribute to IPF.

Pulmonary fibrosis is known to be accompanied by innate and adaptive immune responses; however, the role of inflammation in the disease remains unclear [33]. We propose that L1HS activation and accumulation in AT2 cells might represent an important trigger of the viral cellular sensors and the activation of the innate immune system (macrophages), thereby resulting in the disruption of the immunological balance, which can cause chronic inflammation in IPF. Many of the inflammation-related processes that we associate with TEs have also been previously described as contributing to IPF (IFI6, IFI27, IFI44, OAS1, IL15RA, CX3CL1, and CXCL9) (Supplemental Table 4-C), and they are known to be involved in both fibroblast activation and the accumulation of the extracellular matrix [50]. Furthermore, we confirm some of these processes in our single-cell data analysis, which also shows the highest TE LINE upregulation in the AT2 cells as well as its correlation with some of the genes related to the inflammatory response in IPF patients (i.e. IRF9, STAT1).

The age-related onset of IPF is further evidence that TEs might be involved in IPF and linked to the inflammatory response. Indeed, IPF is most prevalent in individuals aged 60 years or older, and it is partially mediated by senescent cells [21, 51]. The upregulation of intergenic L1HS that positively correlates with SASP markers (i.e., MMP3 and SERPINE1) in AT2 cells suggest that cellular senescence in IPF might be important for L1HS activation. One of the major triggers of cellular-senescence-associated inflammation is the activation of L1 TEs by the age-related loss of epigenetic marks [6, 52]. In accordance with these findings, our study shows the significant upregulation of FOXA1 TF that can bind to the demethylated L1 promoter and thus induce L1HS expression (Fig. 2b-c, Supplemental Table 4B-C). This could potentially result in the activation of the cytosolic sensors for ssDNA (i.e., IFI16), which are responsible for signaling through the cGAS–cGAMP–STING pathway and could induce both interferon-related changes and age-associated inflammation [6, 7]. Indeed, numerous INF-I related genes were upregulated and associated with L1HS expression in IPF cells (Supplemental Table 4B-C). This supports the previously reported importance of cellular senescence, and inflammation in IPF AT2 cells and their potential interaction with L1HS upregulation [25, 53]. We expect future work to uncover additional mechanisms involved in TE activation in IPF.

### Expression of TE loci is associated with expression of IPF related genes

The inflammatory response is not the only outcome of uncontrolled global TE expression, as the activity of individual TE loci can also play an important role in modulating the expression of adjacent genes [48]. Our data show that the TE expression in the vicinity of several genes is associated with the gene expression in both the AT2 and AM cells in the IPF patients. Notably, some of these genes have previously been associated with IPF. For example, the MUC5B promoter variant is one of the major IPF risk factors associated with an increase in gel-forming mucin, which produces mucosal host defensive dysfunction in the bronchi and so is critical in IPF [54]. Our observation that TEs might be involved in the regulation of this gene is based on the significantly higher expression in IPF patients noted from TEs embedded in the intronic region of the gene. Other examples relevant to TEs include the CHL1 gene, which based on previous studies exhibits > 0.8 specificity and 0.9 sensitivity in distinguishing IPF patients from healthy controls, meaning that it is a potential IPF drug target [55]. Although no studies have yet related TEs in CHL1 with IPF, previous studies have proposed that the L1P6 TE within CHL1 can act as an L1 antisense promoter and so drive the transcription of chimeric transcripts [56]. Our study also shows the upregulation of L1P6 to explain ~ 25% of the CHL1 expression, which indicates that this gene might potentially play a regulatory role in relation to CHL1 and so contribute to IPF. One of the fundamental genes associated with the control of proteolysis (SERPINB3) [34], together with the GSTA2 gene, which has previously been associated with IPF [57], show potential regulation from TEs outside the gene. Both these genes have TEs located in close downstream proximity (within 2 kb) to the gene, and while they were not formerly described in other studies, they might have an AT2 cell-specific regulatory function. Although the majority of TE changes are identified in the AT2 cells, the AM cells also show TEs association with metalloproteases (MMP7 and 14), interleukin (IL15RA), chemokine (CCL22), and osteopontin (SPP1), which are all implicated in the development of IPF [45].

While expression of these immune-related genes (e.g., CCL22, IL15RA) is correlated with locus-specific TE expression in AM cells, their regulation can also be a consequence of downstream regulation caused by inflammation in AT2 cells. The AM are also crucial players in respiratory system defense that involves the absorption of harmful particles and infectious agents [58]. Such processes could trigger genome instability (i.e. demethylation) and also directly activate TEs and their adjacent genes in AM.

The TE locus expression changes in our study that are associated with the adjacent gene expression are mainly located in either the 3’UTR, intronic, or 5’UTR region of the respective genes. Previous studies have shown that TEs can be found in gene regions and impact gene regulation by acting as, or interfering with, the regulatory elements in different tissue [59]. In particular, the SINE (Alu) elements that we identify as commonly embedded and expressed in the 3’UTR region are preferentially located in gene-rich regions due to their size (300 bp). The embedded Alu sequences can regulate the translation of their host genes acting as cis elements, or they can be involved in the microRNA regulatory network and many other regulatory process [60]. The role of these sequences in regulating the genes within lung cells as well as their relationship with the IPF disease have yet to be determined.

TEs are sometimes retained within introns and transcribed before the transcripts are processed, or else they are sometimes not spliced out at all, as indicated by a detailed study of the LINE elements [61]. The TEs retained within introns might result from non-preprocessed RNA and so might represent transcriptional noise rather than a biological signal. Thus, it must be noted that our study has limitations related to the quantification of authentic, independently transcribed TE loci.

The significant association that we have identified do not inevitably mean that a given TE indeed has a dominant effect on the transcription of the nearby gene. Transcriptional change of a given gene could also affect the expression of the nearby TEs, or TEs might not have an effect on gene expression at all as gene expression might be the result of downstream regulation [38, 39]. Thus, functional validation of identified candidate loci should be performed. Aside from these limitations, we could detect specific signals in the AT2 and AM cells that might be relevant to IPF and that were repeated in the single-cell data analysis. In addition, although only a small number of patients were examined, our profiling of the three cell types obtained from the same patients suggests the strongest signal to occur in the AT2 cells, which are known to be injured in IPF. This study, therefore, identifies numerous candidate loci for further functional studies.

Finally, smoking has been identified as an important risk factor for the development of IPF [62]. Interestingly, our GO term analysis of the TE loci-associated gene expression highlighted the enrichment of a few genes (eight out of 111, *p* < 0.0178) from an earlier study [63]. This earlier study compared the gene expression of the small airway epithelium (SAE) cells of smokers and non-smokers, and it found that the eight genes (i.e., GSTA2, ITGA2, KRT19, MS4A8, MUC20, MUC5B, SCGB1A1, and SNTN) seen to correlate with TE expression in our current study were also dysregulated in smokers. The SAE is the primary area where the early appearances of the majority of smoking-induced lung diseases are noted [64]. Thus, our observation might suggest that oxidative stress from cigarette smoke also represents an important TE-activating agent [65], and further, that it could be important during the very early stage of IPF development, a hypothesis that should be further tested.

## Conclusion

Taken together, our findings suggest a strong link between TE expression and processes known to be key to IPF. Yet, the extent to which the dysregulation of TEs drives IPF, or whether TE activation represents a side effect of pathogenesis, remains unclear. It is, however, tempting to speculate that a combination of TE demethylation due to aging (loss of epigenetic marks) and injury (oxidative stress) accompanied by dysfunctional autophagy can lead to perpetual inflammation and to changes in locus-specific gene expression, which might play a critical role in the development of IPF in genetically predisposed individuals. In addition, our findings indicate potential new venues for therapies. Moreover, they call into question the role of TEs in other lung conditions caused by injury and inflammation (e.g., chronic obstructive pulmonary disease).

## Materials and methods

### Data

A detailed description of the samples has been previously published and is provided in Supplemental Table 1 [18]*.* We used samples from 14 donors and eight pulmonary fibrosis patients for which bulk RNA sequencing data concerning the AT2, AM, and WL cells were available. Briefly put, RNA was extracted and RNA-seq libraries were prepared using a poly(A) enrichment (NEBNext Ultra RNA, New England BioLabs) and sequenced as single-end 75 base pair reads (Illumina NextSeq 500). We also analyzed single-cell data set from separately obtained eight donors and eight IPF patients from the same study. This data was generated using 3′ V2 chemistry kit on Chromium Single cell controller (10x Genomics) and sequenced (Illumina HiSeq 4000). All the methods used for sample processing and sequencing have previously been described [18].

### Quantification of the gene expression and TE activity using bulk RNA-seq

The raw sequencing data were downloaded from the dbGAP database (phs001750.v1.p1) and then processed using the *SQuIRE* set of tools, which integrates the alignment and expression counts for the gene expression and TE expression [26]. We mapped the raw RNA-seq reads to the GRCh38/hg38 (Dec. 2013 release) version of the human genome assembly, which was downloaded from the UCSC Genome FTP site (ftp://hgdownload.cse.ucsc.edu/goldenPath/hg38/bigZips/chromFa.tar.gz), and determined the transcriptional changes based on both the TE family and TE locus. To confirm the observed changes with the *SQuIRE*, we also quantified the TE expression using the *REdiscoverTE* set of tools (http://research-pub.gene.com/REdiscoverTEpaper/) [20]. *REdiscoverTE* was also used to determine the transcriptional changes on the basis of the TE family separated by genomic region (intronic, exonic, and intergenic). Any potential differences in the number of reads across different cell types (WL, AM, AT2) were accounted for by calibrating TEs and gene expression matrices with total counts of gene expression prior to differential gene expression analysis [20].

The advantage of *REdiscoverTE* is that it can specifically model autonomous TE expression. To compare the two methods (*REdiscoverTE* and *SQuIRE*) for genomic-region-level analysis, we used *REdiscoverTE*-based annotation for the exonic, intronic, and intragenic regions and added up the reads obtained by *SQuIRE* per individual locus. This allowed us to compare the differential expression of the aggregated TE families in the genomic regions for the two methods. For both of these TE quantification methods, we used the same downstream approaches to determine the differentially expressed TE families as decribed  below.

The counts were normalized between the samples, and the differential TEs expression between the healthy donors and the IPF patients in AT2, AM and WL cells was determined using the *DESeq2* package in R. Significance of the TE expression was further determined using the Benjamini and Hochberg (BH)-adjusted *p*-value [66]. The volcano plots were constructed using the *ggplot2* function in R.

To identify the genomic locations of the differentially expressed TE loci in the cells, we downloaded the bed file annotation for the 3’UTR, 5’UTR, intron, and exon regions for each individual gene from the UCSC database table [67]. We further intersected the TE bed file coordinates of our differentially upregulated TEs with the different genomic regions using the *GenomicRanges* [68] package in R. Lastly, we identified the locations of the intergenic regions (those without overlap in 3’UTR, 5’UTR, intron, and exon regions) of the differentially expressed TEs and their distance from the nearest genes using the BEDTOOLS function *closest* [29].

### Association between gene expression and TE expression

We first correlated relationship between the L1HS subfamily and the differentially expressed TE-related genes (manually curated list of genes including type I interferons (IFN-I, 84 genes), senescence-associated secretory phenotype (SASP) genes (85 genes), L1 transcriptional regulator genes (two genes), and L1 post-transcriptional regulator genes (17 genes) [6, 31] using linear model (*lm*) function in R. The difference between the IPF and the donor groups for this relationship was further tested using F-test with *aov* function in R where TE subfamily expression represented the independent variable, and gene expression the dependent variable. Phenotype (IPF/donor) was also included in the model as a covariate.

To determine whether the TE expression is related to the adjacent gene expression, we tested the correlation between the TEs located in the intergenic regions and those located within the 5’UTR, 3’UTR, and intron regions using a linear model (*lm*) in which the TE expression in each locus was modeled as the independent variable and the gene expression as the dependent variable, accounting for the phenotype (IPF or donor) as a factor. The TEs that overlapped with the exons or that were located within the exon-intron, exon-5’UTR, or exon 3’UTR regions were excluded from these analyses. We only tested the cis TE-gene pairs, as most of the TEs were either within 50 kb of the gene or within gene regions. We defined the significant TE-gene pairs using the multiple test correction, BH-adjusted *p*-value (*p*-value <0.05) [66]. The same tests were performed for the AT2, AM, and WL data. Difference between the the regression slopes in groups (IPF or donor) was determined using F-test with *aov* function in R. Results were plotted using *ggplot2* function in R.

When the expression of more than one TE adjacent (within 50 kb distance) to the gene of interest was associated with the gene expression, we calculated the relative importance of each TE using the R package *relaimpo* and 1000 bootstraps [69]. We further tested for the gene ontology enrichment of the genes that were associated with TE expression using the STRING database [40].

### Detection of TE activity by means of scRNA-seq

#### Single-cell transcript mapping

The raw reads of the single-cell RNA-seq data were downloaded from dbGAP (phs001750.v1.p1) [18].

The TE annotation library was downloaded using the *SQuIRE* package in R and the GRCh38/hg38 (Dec. 2013 release) genome, together with the TE reference. The genome-TE reference was built and the reads were mapped to both the genes and TE subfamilies using Chromium 10x workflow and the Chromium 10x Cell Ranger Single-Cell Software Suite (http://software.10xgenomics.com/single-cell).

The subfamily of TEs represents groups of sequences from the TE family (LINE, SINE, and LTRs) that are sufficiently distinct in terms of their repeats to allow for unique read mapping. Only the unique alignments were considered and counted for the differential expression analysis. The number of differences is defined as the number of TE subfamilies. The cell tags were matched with the published data matrix so that the same cells were used as in previously published accounts [18]. The filtered feature matrices counts produced by the cell ranger were used in the subsequent analysis using the R package *Seurat* [70].

#### ScRNA-seq clustering

The downstream single-cell analysis was performed using functions in both the *Seurat* package V3 and R 3.5 [70]. First, we matched the cells of eight healthy donors and eight IPF patients from our alignment counts to the published filtered dataset available at GEO (GSE122960) [18]. In doing so, we recovered a total of 77,517 cells. To compare the expression data from different patients and different lung cell types, we integrated the data with the *IntegrateData* function and identified the anchors between the dataset using the *FindIntegrationAnchors* function. The normalization of the 22,009 identified genes was performed based on the total number of unique molecular identifiers (UMIs) per cells, multiplied by a scale factor (10,000) and then log transformed. We next conducted a principal component analysis (PCA) of the top 2000 variable genes and then used the first 20 PCA components to project the cells onto a two-dimensional map using the uniform manifold approximation and projection (UMAP) dimensionally reduction method offered by the *RunUMAP* function. To identify the cell types from the lung tissue, we clustered all the IPF patients and donor cells using the K-nearest neighbor (KNN) graph-based clustering algorithm and the *FindNeighbors* function. Finally, we used the *FindClusters* function (resolution parameter = 0.5) to establish the cell clusters, while the cluster identity was determined based on the conventional cell marker genes [18]. The differential gene and TE subfamilies expression analysis between the IPF patients and the donors was performed for each cell type using the non-parametric Wilcoxon rank-sum test and the Bonferroni correction. In addition, we compared the IPF patients to the donors in terms of the total expression of the L1 TE subfamilies per cell (L1 score) using the *addModuleScore* function and the Wilcoxon rank-sum test. To calculate the L1 score per cell, we used the average expression of 70 L1 subfamilies. We next tested whether the relationship between the L1 score and the TE related genes (123 expressed genes out of 188 genes from four gene sets known to be part of the L1 defense and antiviral interferon-stimulating genes). A linear model was fitted for each L1 score and the gene pairs for the IPF patients and the donors using the *lm* function in R, while the difference between the regression lines of two groups was determined using the t-test and *aov* function in R.

## Supplementary Information

**Additional file 1: Supplemental Figure 1.** Reproduced changes in the expression of TE families in individual cell types and whole-lung tissue obtained by *REdiscoverTE* method**.** A. Bulk RNA-seq of WL. B. Bulk RNA-seq of AT2 cells. C. Bulk RNA-seq of AM. The TE expression was determined by read counts using the *REdiscoverTE* workflow, and the differential expression analysis was performed using the *DESeq2* package in R. The x-axis represents the log_2_ ratio of the TE subfamily expression between the IPF patients and the donors. The y-axis represents the adjusted *p*-value based on -log_10_. The red color represents the TE subfamilies with a fold expression change (FC) > 1 and p.adjust < 0.05 (BH adjusted) (as represented by two vertical dotted lines, and the horizontal line). The blue color represents the TEs with an adjusted *p*-value (p.adjust < 0.05), while the green dots represent the TEs with an FC > 1. The black colors represent changes that do not have a significant p.adjust value or an FC higher than 1. Different shapes represent different TE families.

**Additional file 2: Supplemental Figure 2.** L1HS intergenic expression in Donor and IPF samples as quantified by *SQuIRE* method. Green: donor samples. Red: IPF samples. The x-axis represents groups, while the y-axis represents log normalized expression values for counts from all intergenic L1HS loci (see Materials and Methods). The L1HS between the groups is not significantly different as determined by *DESeq2* analysis (*p* = 0.037, p.adjust =0.11).

**Additional file 3: Supplemental Figure 3.** Feature plot of the expressed cell markers for the cell-type identification in the UMAP plot. The cell types are classified as epithelial cells (alveolar type II cells (AT2), alveolar type I cells (AT1), ciliated cells, basal cells, and club cells), immune cells (alveolar macrophages (AM), monocytes, B cells, plasma mast cells, dendritic cells, and T cells), and mesenchymal cells (fibroblasts and endothelial cells).

**Additional file 4: Supplemental Table 1.** Summary of the samples used in the bulk RNA-seq and scRNA-seq analyses. Each row represents the sample ID, while the columns indicate the histological types, age, sex, race, smoking history, and phenotype, as reported in the original publication [18]. All the fibrosis samples are grouped and analyzed together, as indicated in the table (analyzed phenotype. HP: hypersensitivity pneumonitis; ILD: interstitial lung disease; IPF: idiopathic pulmonary fibrosis; NA: not available; SSc: systemic sclerosis. An additional sample description can be obtained from the original manuscript.

**Additional file 5: Supplemental Table 2.** Summary of the differential analysis of the TE families between the IPF patients and the donors in different cells based on *SQuIRE* and *REdiscoverTE* analysis. A. Whole-lung (WL) tissue - *SQuIRE*, B. Alveolar type II (AT2) cells - *SQuIRE*. C. Alveolar macrophages (AM) - *SQuIRE*. D. Whole-lung (WL) tissue - *REdiscoverTE*, E. Alveolar type II (AT2) cells - *REdiscoverTE*. F. Alveolar macrophages (AM) - *REdiscoverTE*. The columns represent the repeat classification, the TE expression base mean (baseMean), log_2_ fold change (log2FoldChange), lfcSE (log fold standard error), stat (Wald statistics; lfc/standard error), *p*-value, p.adj (BH adjusted *p*-value). Only significant upregulated families are shown. G. Summary of all changes in *REdiscoverTE* analysis.

**Additional file 6: Supplemental Table 3.** Summary of the differential analysis of the TE families between the IPF patients and the donors in AT2 cells based on genomics regions and *SQuIRE* and *REdiscoverTE* analysis. A. Differential TE expression in exonic region in AT2 cells – *RediscoverTE*. B. Differential TE expression in intronic region in AT2 cells – *RediscoverTE*. C. Differential TE expression in intergenic region in AT2 cells– *RediscoverTE*. D. Summary of all changes obtained by *SQuIRE* and *REdiscoverTE.* E. Differential TE expression in intergenic region in AT2 cells- *SQuIRE.* Only significant TE families are shown. The columns represent the repeat classification, TE expression base mean (baseMean), log_2_ fold change (log2FoldChange), lfcSE (log fold standard error), stat (Wald statistics; lfc/standard error), *p*-value, p.adj (BH adjusted *p*-value).

**Additional file 7: Supplemental Table 4.** Summary of the differential analysis and correlation between the L1HS and their functionally related genes in AT2 cells. A. Manually curated list of genes known to be part of the L1HS defense and antiviral interferon-stimulating genes [25, 39]. B. List of all significant differentially expressed genes in AT2 cells, with functional annotation of L1HS related genes. Only significant genes are shown. The columns represent the gene names, gene expression base mean (baseMean), log_2_ fold change (log2FoldChange), lfcSE (log fold standard error), stat (Wald statistics; lfc/standard error), *p*-value, p.adj (BH adjusted *p*-value). C. Summary of the linear model test of association between the expression of L1HS -related genes and L1HS. The *p* value^1^ represents relationship between gene expression and L1HS-locus expression, and the *p* value^2^ - represent difference in the regression slopes between the two groups (IPF and donor). The *p*-value correction was performed using the Benjamini and Hochberg (BH) test and (p.adjust^1^ and p.adjust^2^). Model: Gene expression∼L1HS expression + group (IPF/Donor).

**Additional file 8: Supplemental Table 5.** Summary of the differential analysis of the TE loci and genes between the IPF patients and the donors in different cells. A. Whole-lung (WL) tissue, B. Alveolar type II (AT2) cells. C. Alveolar macrophages (AM). The columns represent the gene expression base mean (baseMean), log_2_ fold change (log2FoldChange), lfcSE (log fold standard error), stat (Wald statistics; lfc/standard error), *p*-value, p.adj (BH adjusted *p*-value). The plus and minus signs next to the gene names indicate the gene orientation.

**Additional file 9: Supplemental Table 6.** Summary of the linear model test of all the identified TE-gene loci pairs. A. AT2 linear model analysis summary. B. AM linear model analysis summary. The association between the log_2_-transformed TE expression (independent variable) and the log_2_-transformed gene expression (dependent variable) was tested using a linear model. Genes, TE locus, distance from the gene and the genomic region of the TE locus are shown. The *p* value^1^ represents relationship between gene expression and adjacent TE-locus expression, and the *p* value^2^ - represent difference in the regression slopes between the two groups (IPF and donor). The *p*-value correction was performed using the Benjamini and Hochberg (BH) test and (p.adjust^1^ and p.adjust^2^).

**Additional file 10: Supplemental Table 7.** Summary of the differential analysis of the genes and TEs per individual cell type in the scRNA-seq. Table 5A. Cell number for each subject in each cell type. B. Differentially expressed genes in IPF vs Donor for all cell types. Avg_logFC: Average log fold change; *p*-value: calculated based on the Wilcoxon rank-sum test; p.adjust: adjusted *p*-value based on the Bonferroni correction using all the genes in the dataset; pct.1: fraction of the cells that express the gene in IPF patients; pct.2: fraction of the cells that express the gene in donors. C. Differentially expressed TE families in the IPF patient vs. donor samples for the different cell types. D. Differences between the IPF patients and donors represented as correlation between L1 score and TE-related gene expression. A subset of 126 genes identified in RNA bulk seq were tested. A linear model was fitted for each L1 score and the gene pairs. The *p*-value was calculated using t-test; p.adjust is the Benjamini and Hochberg (BH)-adjusted *p*-value.

## Data Availability

All data generated or analyzed during this study are included in Reyfman et al. [18] published article and its supplementary information files. Raw genomics reads and detailed patient data are available from the GSE122960 dataset and from the dbGAP database (phs001750.v1.p1), although restrictions apply with regard to the availability due to dbGAP policy. Data access was obtained from dbGAP (Project #21807).
