# Supplementary material for: The derepression of transposable elements in lung cells is associated with the inflammatory response and gene activation in idiopathic pulmonary fibrosis
Source: Mob DNA. 2021 Jun 9;12:14. doi: 10.1186/s13100-021-00241-3 (PMC8191028; doi:10.1186/s13100-021-00241-3)

Supplemental Figure 2

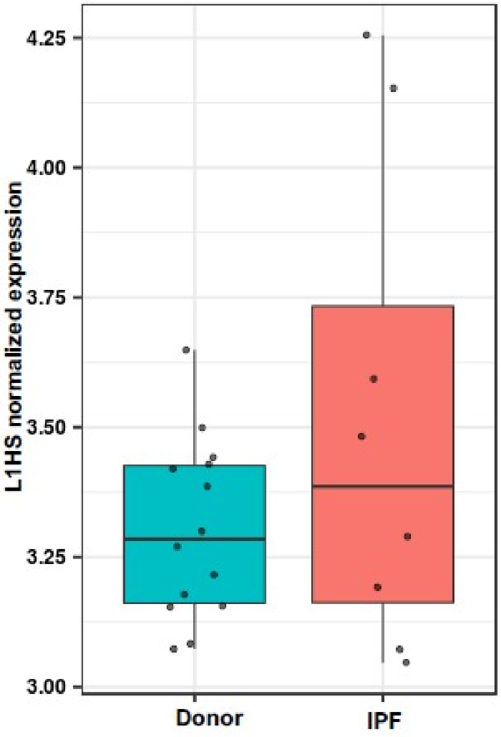

Supplement: Supplementary file 2 [file 13100_2021_241_MOESM2_ESM.docx]
